# Performance Analysis of High-Performance Concrete Materials in Civil Construction

**DOI:** 10.3390/ma16165711

**Published:** 2023-08-21

**Authors:** Yongguang Han, Tianhua Zhou

**Affiliations:** 1 Architectural Engineering College of Chongqing City Vocational College, Chongqing 400044, China; hans0537@126.com; 2 School of Civil Engineering, Chang’an University, Xi’an 710054, China

**Keywords:** civil construction, high-performance concrete, mechanical properties, durability, building materials, corrosion

## Abstract

This paper develops the mechanical and durable samples of C50 high-performance concrete, studies the mechanical properties, crack resistance, sulfate attack resistance, frost resistance, and impermeability of concrete with different mineral admixtures of mineral powder and fly ash, and obtains the best mineral admixture of mineral powder and fly ash to improve the performance of high-performance concrete. The results show that the doping effect is the best when the ratio of prepared mineral powder to fly ash is 3:2. With the increase in the mineral powder–fly ash admixture, the slump and expansion of high-performance concrete decrease rapidly at first and then slowly. In total, 60% doping is the turning point; the compressive and flexural strengths of concrete decreased slowly at first and then rapidly. Taking 30% of the admixture as the turning point, 35% of the mineral powder fly ash is generally selected. By mixing and adding a certain proportion of fly ash and mineral powder admixtures, the crack resistance of concrete is enhanced, and the shrinkage and cracking are reduced. The corrosion resistance coefficient will exceed 88%, the relative dynamic elastic modulus will exceed 95%, and the impermeability grade will reach P17. The durability of concrete can be improved by adding mineral admixtures.

## 1. Introduction

In civil engineering, the proper selection of construction materials is crucial to the development of the project. With the development of science and technology, housing construction will use a variety of materials to improve performance [1]. In the face of increased demand, the wide application of high-performance concrete promotes the development of modern construction in the direction of bigger, stronger, and more environmentally friendly buildings. High-performance concrete has high strength and can replace traditional concrete with economic and social benefits [2]. The deterioration of the engineering environment and chemical corrosion has increased the impact on high-performance concrete. Various destructive factors reduce the stability and durability of concrete components, delay service life, and even cause safety hazards. Therefore, to cope with the effects of freezing and thawing, chemical corrosion, etc., the study of high-performance concrete with excellent durability has become an international research hot spot [3].

The aim of this study is to analyze the characteristics of high-performance concrete and find the optimal mineral powder–fly ash composite admixture to provide data support for the selection of concrete for civil construction, provide a reference for practitioners and decision-makers, and also provide insights for future research directions. By analyzing high-performance concrete, the incorporation of mineral admixtures is of significant technological innovation. Although there have been studies on the performance of high-performance concrete in civil construction, there are some shortcomings. Previous studies may have been limited in analyzing high-performance concrete in depth without comprehensively examining its actual performance under different environmental conditions. Practical applications are constrained by cost and preparation processes, and the variations in performance in different environments needs to be studied in depth. Finally, more research is needed on economic evaluation to ensure the long-term viability of high-performance concrete. Although progress has been made, further research is needed to gain a more comprehensive understanding of the application of high-performance concrete in civil construction.

In the study conducted by Guo et al., the thermal treatment and utilization of flue gas desulphurization gypsum as an admixture in cement and concrete were investigated [4]. The potential application of flue gas desulphurization (FGD) gypsum as an admixture in cement and concrete was explored. The abrasion resistance of steel slag concrete and its resistance to chloride penetration was assessed using the NEL method and the ASTM C1202 method, as described by Li et al. [5]. The impact of the admixture on concrete strength and frost resistance was evaluated through mercury intrusion and rapid freeze–thaw methods, maintaining a consistent water/binder ratio, following the approach outlined by Zhang et al. [6]. Zong researched the utilization of compound admixtures in the construction of frozen mine shaft lining concrete, providing data for optimizing the mix proportion of concrete admixture content [7]. He et al. employed ASTM C1202 to investigate the effects of mineral admixtures on the resistance of chloride ions penetration in the new concrete compound system [8]. Li et al. employed ring-test methods to study the early-age cracking characteristics of compound mineral admixture concrete, incorporating steel slag, blast furnace slag, and fly ash [9]. Yu et al. addressed the global permeability issues encountered in ordinary cement concrete (OCC), which negatively impact construction and infrastructure by reducing the lifespan and increasing maintenance costs [10]. They proposed an improvement strategy for OCC impermeability by introducing a novel compound admixture consisting of four chemical admixtures, filling agent (F), water-reducing agent (WR), fluorosilicate-based agent (FB), and expansion agent (E), blended into the fresh concrete. Lu et al. investigated the influence of an alcohol–amine compound rust inhibitor on concrete properties using commonly employed mineral admixtures, including slag, fly ash, and silica fume. The study focused on evaluating the effects of this rust inhibitor on the compressive strength, chloride penetration resistance, carbonation resistance, and rebar corrosion resistance of concrete formulations, incorporating a significant proportion of mineral admixtures [11]. The addition of silica fume to enhance the strength of high-performance concrete also brings about significant self-drying and shrinkage issues, accompanied by an increasing water demand that fails to mitigate temperature concerns. Grinding slag helps reduce water consumption, yet it leads to drying and shrinkage. Incorporating fly ash mitigates self-drying shrinkage and reduces water requirements, albeit at the cost of compromised carbonation resistance [11,12,13]. Researchers have effectively employed the synergistic effect of multiple mineral admixtures in concrete, achieving enhanced results [14,15,16]. Concrete stands as the cornerstone material in construction, with diverse large-scale projects demanding heightened concrete structure and performance criteria. As the engineering environment grows more complex and demanding, concrete performance faces elevated expectations, and optimizing high-performance concrete materials allocation poses a significant challenge for civil engineering [17]. The compressive strength serves as a metric for evaluating the mechanical properties of fly ash concrete [18]. This study centers on high-performance concrete, analyzing its attributes, conducting mechanical and durability tests on C50 high-performance concrete, and determining the optimal mineral powder–fly ash composite admixture ratio at 35%. The core objective of this research lies in exploring concrete doping characteristics, thereby offering theoretical support to enhance high-performance concrete’s overall performance.

## 2. Materials and Methods

### 2.1. Characteristics of High-Performance Concrete

High-performance concrete has many performance advantages, and its application in civil engineering will greatly improve the quality of civil engineering and promote the sustainable development of civil engineering. The most important advantage of high-performance concrete is its excellent compactness and impermeability, and its fluidity and segregation resistance are better [19]. When preparing high-performance concrete, the elastic modulus should be set at 30~55 GPa, and the drying shrinkage rate should be less than 0.04% to ensure the stability of its volume, which is not easy to change and makes high-strength and high-performance concrete play an excellent role [20].

The main factors that affect the performance of concrete are water–binder ratio and cementitious materials. By adding various mineral admixtures to concrete, the water–binder ratio and the dosage of cementitious materials can be adjusted to realize the influence on workability and mechanical properties in civil engineering [21]. High-performance concrete can meet the practical requirements of civil construction by adding minerals and admixtures.

### 2.2. Test Methods

In this paper, different water–binder ratios, the dosage of cementitious materials, the dosage of additives, and mineral admixtures of C50 high-performance concrete will be used to analyze the changes in the mechanics and durability of high-performance concrete. The properties of high-performance concrete include mechanical properties, crack resistance, frost resistance, impermeability, and so on.

The mechanical property test of high-performance concrete will be carried out according to the cement concrete test regulations. The specimen used in the unconfined compressive strength test was a 15 cm × 15 cm × 15 cm cube, the curing period was set to the specified age test, and the load was increased at a speed of 0.5~0.8 MPa/s. A cuboid with a size of 15 cm × 15 cm × 550 cm was used for the flexural strength test, and its curing period was set to a specified time.

The early crack resistance experiment was evaluated by the early cracking of concrete. In the test, the specimens were set to different curing periods of temperature, humidity, and time, and the test environment was set to temperature, humidity, windless, and windy conditions. A specimen of 60 cm × 6 cm × 5.3 cm was used in the test, and steel bars were used in the mold to restrain the contraction of the flat plate. In total, 2 h after the specimen was formed, the concrete surface was blow dried at a wind speed of 0.8 m/s, and the cracking time and the number of cracks of the specimen were recorded until the time reached at least 24 h.

Sulfate corrosion resistance test was mainly based on the standard of long-term performance and the durability test method of ordinary concrete. Samples were made of 10 cm × 10 cm × 10 cm concrete samples, which were divided into 5 groups. In the test, two days before the curing of the samples, dry the samples to be cured and put them in a 90 °C oven, bake them for 24 h until they are dried, then naturally cool them to room temperature in a dry environment, and put them in a box of samples. Pour the 5% Na_2_SO_4_ solution with a temperature of 25–30 °C into and submerge the surface of the specimen for at least 20 mm. After soaking, empty the solution and air dry it within half an hour. After air drying, raise the temperature in the specimen box to 90 °C and bake it for 6 h. After drying, cool to room temperature, repeat drying and wetting cycles several times, and then test the compressive strength of the sample.

The frost resistance test was mainly based on the test regulations of cement and cement concrete for highway engineering. The specimen is 10 cm × 10 cm × 40 cm, and the freeze–thaw cycle tests of 50, 100, 150, 200, 250, and 300 times were carried out to measure its relative dynamic elastic modulus.

For the impermeability test, according to the long-term performance and durability test method of ordinary concrete, six cone-shaped specimens with upper diameters of 175 mm, lower diameters of 180 mm, and heights of 150 mm were prepared. The initial pressure was set to 0.1 MPa in the test, and 0.1 MPa water pressure was added every 8 h. When 3 of the 6 cone-shaped specimens had water under pressure overflowing, the test was stopped.

The experimental sequence of this paper was to make experimental concrete samples with different doping ratios according to the experimental needs. Firstly, its mechanical properties (compression resistance and flexural resistance), crack resistance, frost resistance, and impermeability were tested, in turn, to optimize the optimal doping ratio.

The vibrating table shall conform to the relevant provisions of the current industry standard “Vibrating table for concrete test” IG/T245, the vibration frequency shall be 50 Hz and 2 Hz, and the vertical amplitude of the center point of the vibrating table shall be 5 mm and 100 mm under no load. The tamper should comply with the relevant provisions of the current industry standard “Concrete slump meter” JG/T 248, with a diameter of 16 mm and a length of 600 mm.

At 5 mm, the end should be hemispherical. The hammerhead quality of the rubber hammer or mallet should be 0.25 kg~0.50 kg. Before the specimen is formed, the size of the test mold shall be checked and shall comply with the relevant provisions of this standard; wipe the test mold clean, and evenly coat a thin layer of mineral oil or another isolation agent that does not react with concrete on the inner wall of the test mold. The isolation agent on the inner wall of the test mold should be evenly distributed without evident deposition. Before the concrete mixture enters the mold, it should ensure its homogeneity. According to the consistency of the concrete mixture or the purpose of the test, the appropriate molding method should be determined, and the concrete should be fully dense to avoid delamination and segregation.

## 3. Results

### 3.1. Mechanical Characteristics of High-Performance Concrete Materials

In the mechanical experiments of high-performance concrete materials, the proportion of mineral admixtures determines the performance of concrete. In this paper, 60% mineral powder and 40% fly ash are selected, according to the compressive strength, flexural strength, and detailed inspection of the percentages of different components [22]. The mineral powder fly ash composite mineral admixture shall be prepared according to the proportion of mineral powder:fly ash = 6:4. The influence of composite mineral admixtures of different quality on the mechanical properties of concrete shall be carried out, and the optimal range of admixture shall be determined to obtain high-performance concrete with high mechanical properties.

During the test, the mixing amounts of composite mineral admixtures were set as 0%, 15%, 30%, 45%, and 60%, respectively. The mixing amount mainly referred to the proportion of total cement and mineral admixtures. Table 1 shows the mix proportions of different amounts of mineral powder fly ash composite mineral admixtures. The total amount of cementitious materials is 470 kg, the water binder ratio is 0.33, and the sand ratio is 38%. Concrete pouring temperature should be determined according to local meteorological conditions and concrete construction needs. Generally speaking, the concrete pouring temperature should be between 5 °C and 35 °C. If the temperature is lower than 5 °C, the setting time of concrete will be prolonged, resulting in the decrease in concrete strength; if the temperature is higher than 35 °C, the concrete will set too fast, and it is easy to crack and deform.

**Figure 1:**
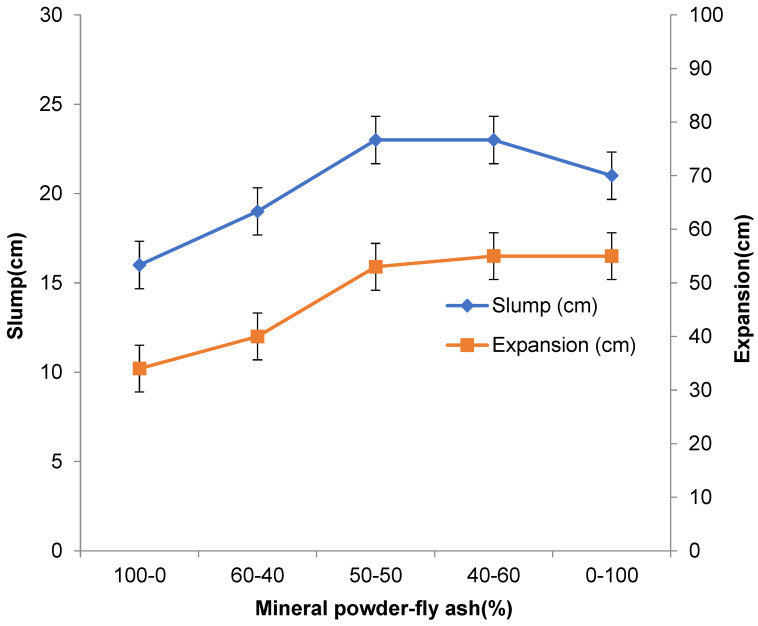
Influence of compound mineral admixture amount on the workability of concrete.

**Figure 2:**
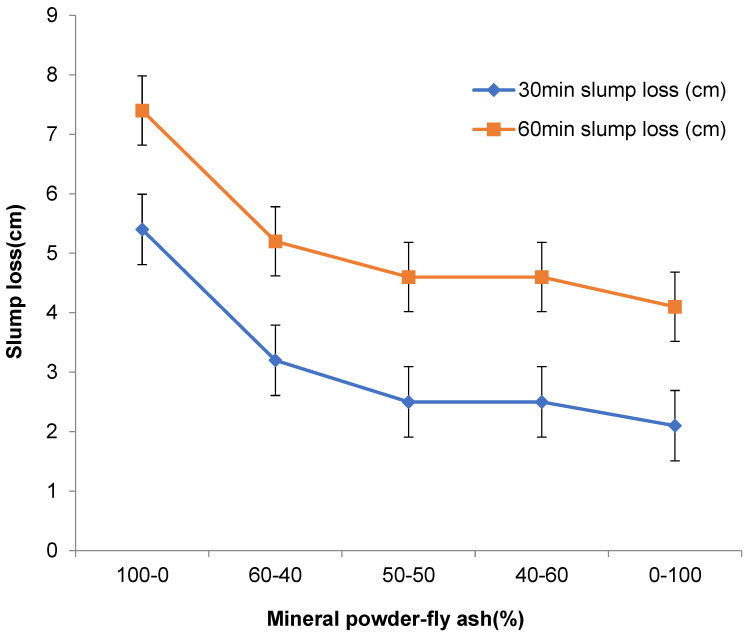
Influence of compound mineral admixture amount on concrete slump loss.

In this experiment, the mechanical properties of concrete with different mineral admixtures of mineral powder and fly ash were tested. For the experimental method, refer to GB/T 50082-2009’s Test Method for Durability and Long-term Mechanical Properties of Concrete. Table 2 shows the influence of compound mineral admixture amount on concrete mechanical properties. Figure 1 shows the influence of compound mineral admixture amount on concrete slump and expansion, and Figure 2 shows the influence of compound mineral admixture amount on concrete slump loss.

From the figures and tables, it can be seen that the mineral powder–fly ash composite admixture with different dosages has an evident influence on the mechanical properties of concrete. When the dosage of composite mineral admixture increases gradually, the slump and expansion of high-performance concrete will increase rapidly. When the dosage reaches more than 50%, the concrete will show greater fluidity, and the slump and expansion of the concrete will increase slowly when the dosage exceeds 70%. With the increase in the amount of composite mineral admixture, the slump loss of concrete decreases rapidly at first and then slowly, and the amount of 60% is the turning point.

**Figure 3:**
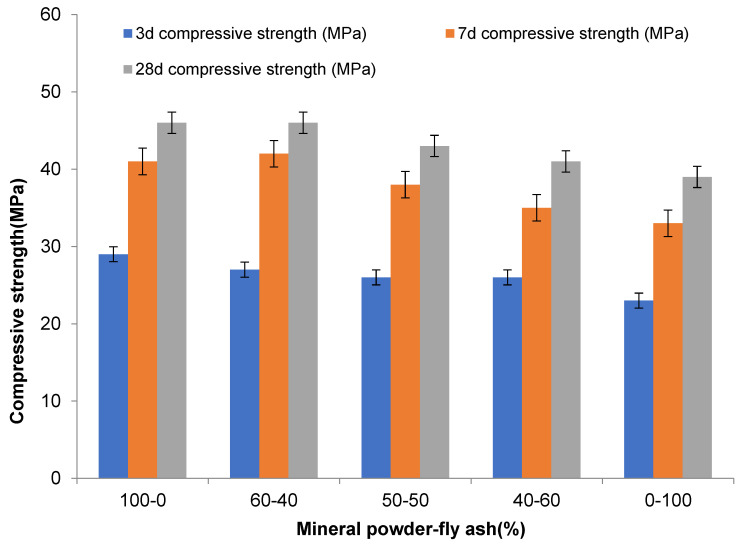
Influence of compound mineral admixture amount on concrete compressive strength.

**Figure 4:**
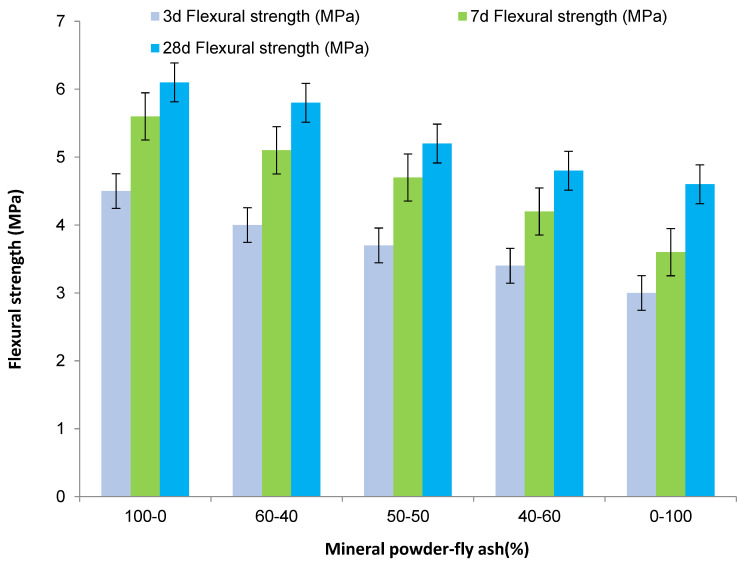
Influence of compound mineral admixture amount on flexural strength of concrete.

The compressive strength and flexural strength of composite mineral admixtures with different amounts were obtained through experiments. Table 3 shows the influence of composite mineral admixtures on the mechanical properties of concrete. Figure 3 shows the influence of the amount of composite mineral admixture on the compressive strength of concrete. Figure 4 shows the influence of the amount of composite mineral admixture on the flexural strength of concrete.

It can be seen from the figures and tables that the compressive and flexural strength of concrete decreases slowly at first and then quickly with the continuous increase in the amount of composite mineral admixture. When the amount is 0~30%, the strength of concrete has no evident change. When the amount of admixture exceeds 30%, the compressive strength and flexural strength of concrete will decline rapidly. This is mainly because the hydration of cement increases the activity of mineral powder and fly ash in the composite mineral admixture gradually, which promotes the secondary hydration reaction of free calcium oxide and highly alkaline C-S-H and improves the strength of concrete. Therefore, the composite mineral admixture of C50 high-performance concrete should be 30–45%. Considering the stability of concrete performance and economic cost, the amount of mineral powder–fly ash composite mineral admixture is generally 35%.

**Figure 5:**
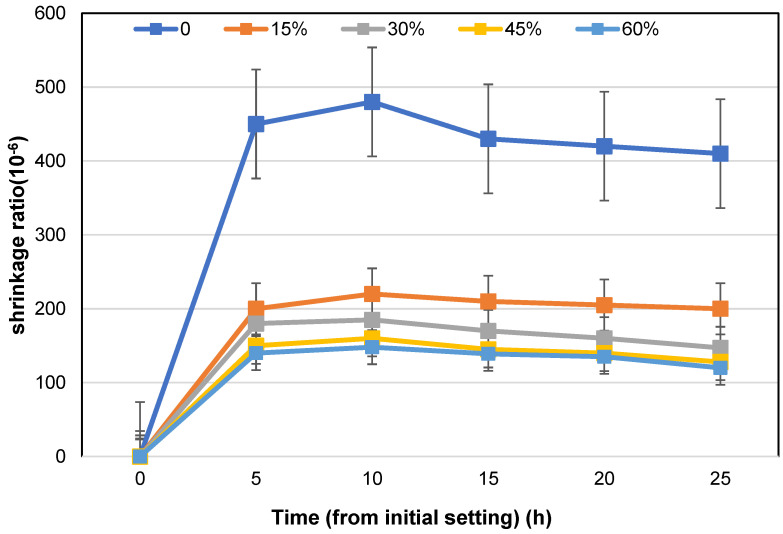
Influence of mineral powder content on shrinkage of concrete under sealed condition within 1 d.

It can be seen from Figure 5 that the self-drying shrinkage also decreases with the increase in mineral powder content, and the larger the mineral powder content the smaller the shrinkage. It shows that the addition of mineral powder has a significant inhibitory effect on the self-drying shrinkage of high-performance concrete with a low water–binder ratio, and this inhibitory effect is more evident with the increase in mineral powder content.

### 3.2. Durability Characteristics of High-Performance Concrete Materials

The crack resistance, sulfate attack resistance, frost resistance, and impermeability of C50 high-performance concrete are studied through experiments. The shrinkage-cracking model is used to quantitatively analyze the early cracking model of concrete, and the shrinkage crackings of different types of concrete are predicted by the model. The main factors leading to concrete shrinkage cracking are temperature deformation, shrinkage deformation, drying shrinkage deformation, elastic modulus, tensile strength, etc. [22]. These factors are all related to time and change dynamically with time. The influence of mineral admixtures on the crack resistance of high-performance concrete was studied by a plate restraint test. Table 4 shows the cracking of C50 high-performance concrete for 24 h. From Table 4, it can be seen that mixing a certain proportion of fly ash and mineral powder admixture can directly improve the early-age crack resistance of high-performance concrete compared with the concrete without or only with fly ash or mineral powder. By adding fly ash, the crack resistance of concrete is enhanced, shrinkage is reduced, and cracking is reduced, but the effect of adding mineral powder is not evident.

In this paper, the change in compressive strength of samples after the wet–dry cycle is used to characterize the damage to concrete. Table 5 shows the sulfate attack resistance of C50 high-performance concrete. It can be seen from Table 5 that the sulfate resistance of concrete has been significantly improved after adding mineral admixtures compared with the reference sample. At the same time, the corrosion resistance of concrete mixed with fly ash and mineral powder, at the same time, is better than that of concrete mixed with anyone alone. When composite mineral admixtures are added to concrete, the corrosion resistance coefficients of 7 d and 28 d are above 88%. The corrosion resistance coefficient of concrete with fly ash alone is less than 81%, which shows that the effect of fly ash on improving the sulfate resistance of concrete is not evident. The corrosion resistance of concrete with only mineral powder is more than 80% in the early and late stages, indicating that mineral powder is helpful in improving the sulfate resistance of concrete. Therefore, a certain proportion of fly ash and mineral powder can significantly improve the early and late sulfate resistance of concrete.

For concrete, its frost resistance has a great influence on its durable service life. When civil engineering projects are located in harsh areas, its frost resistance requirements are very strict. In this paper, the relative dynamic modulus of 28 d concrete under different freeze–thaw cycles is tested by experiment. Table 6 shows the relative dynamic modulus of concrete under different freeze–thaw cycles. It can be seen from Table 6 that the frost resistance of high-performance concrete can be significantly improved by adding mineral powder fly ash mineral admixtures compared with those with only or without mineral admixtures. When the freeze–thaw cycles exceed 300 times, the relative dynamic modulus of C50 high-performance concrete will exceed 95%. The relative dynamic elastic modulus of concrete with fly ash only is higher than that with mineral powder, which shows that fly ash has a good effect on improving the compactness and frost resistance of concrete.

The impermeability of concrete is the main cause of chloride ion corrosion on concrete. In this paper, the influence of mineral admixture on the impermeability of concrete is analyzed by testing the impermeability grade of concrete with only one mineral admixture and composite mineral admixture at the age of 28 days. The impermeability of concrete is given in Table 7. It can be seen from Table 7 that the composite mineral admixtures will help to improve the impermeability of concrete, and the grade will reach P17, which fully meets the requirements of civil engineering on the impermeability of concrete. Mixing only one mineral admixture can also effectively improve the impermeability of concrete. The impermeability grade of concrete with only (KF) and only (FMH) reaches P11 and P13, whereas the impermeability of reference concrete is only P5. Therefore, it is effective to improve the impermeability of concrete by adding mineral admixtures.

## 4. Discussions

This thesis aims to investigate the key performance characteristics of high-performance concrete materials in civil construction, including the analysis of compressive strength, durability, crack control, and sustainability, to guide material selection and engineering application based on scientific data for the construction field. Through the comprehensive study of different ratios, admixtures, and construction processes, it aims to deepen the understanding of high-performance concrete and provide feasible suggestions for practical engineering applications.

(1) In this paper, the ratio of mineral powder to fly ash is 3:2, and different amounts are mixed in the concrete. With the increase in the admixture, the slump and expansion of concrete decreased rapidly and then slowly, and the admixture of 60% was the turning point. With the increase in the mineral powder–fly ash composite mineral admixture, the compressive strength and flexural strength of concrete first decreased and then decreased rapidly. When the admixture is 0–30%, the change in concrete strength is not evident. When the admixture exceeds 30%, the compressive strength and flexural strength of concrete decrease rapidly. After mixing mineral powder, the shrinkage of concrete changes greatly in one day, and the larger the dosage the smaller the shrinkage of concrete. Generally, 35% of mineral powder–fly ash composite mineral admixture is used;
(2) A certain proportion of fly ash and mineral powder admixture can directly improve the early crack resistance of high-performance concrete; fly ash can enhance the crack resistance of concrete and reduce shrinkage and cracking, but the role of mineral powder is not evident. When the composite mineral admixture is incorporated into concrete, the corrosion resistance of concrete will be more than 88%, whereas the corrosion resistance of concrete with fly ash alone is less than 81%. After more than 300 freezing and thawing cycles, the relative dynamic modulus of C50 high-performance concrete will be more than 95%, and the relative dynamic modulus of concrete with a single admixture of fly ash is higher than that of concrete with a single admixture of mineral powder. Composite mineral admixture helps to improve the impermeability of concrete, which will reach the grade of P17. Admixture of only one mineral admixture is also effective in improving the impermeability of concrete, which reaches the grades of P11 and P13 for (KF)-only and (FMH)-only concretes. An admixture of mineral admixtures helps to improve the durability of concrete.

Compared to the study of single admixtures, the use of composite admixtures can cover more material combinations, thus providing a more comprehensive understanding of the multidimensional effects of different admixtures on concrete performance and providing a more scientific basis for the application of concrete.

Concrete with a composite admixture of fly ash and mineral powder has multiple advantages. Introducing these auxiliary materials, fly ash and mineral dust, into the concrete preparation process not only significantly improves the strength and durability of concrete but also helps to improve the flow and workability, making construction easier. The silicate and aluminate components of these materials participate in the hydration reaction of cement, enhancing the matrix structure of concrete and effectively inhibiting temperature rises and crack formations. Meanwhile, being environmentally friendly, the reuse of waste helps to reduce resource consumption and achieve sustainable construction. In addition, the addition of fly ash and mineral powder can also slow down drying and shrinkage, reducing the risk of cracking. However, the proportioning should be rationalized according to the specific situation, and the effects of different sources and properties on the performance of concrete should be fully considered to ensure that the concrete meets the design requirements.

However, the source and quality of admixtures will have an impact on the experimental results, and there is a need to ensure the consistency and reproducibility of the experiments. Although the results under laboratory conditions provide insights into the effects of composite admixtures, in actual engineering applications, there are factors such as environment, construction, and durability, which require more practical validation and in-depth research.

## 5. Conclusions

In this paper, C50 high-performance concrete material is taken as the research object; the mechanical properties, crack resistance, sulfate resistance, frost resistance, and impermeability of concrete with different amounts of mineral powder–fly ash composite admixture are studied by analyzing the performance characteristics of the high-performance concrete; and the optimal mineral powder-fly ash composite admixture is obtained, which provides theoretical data support for improving the performance of high-performance concrete. The main research results are as follows.

Experiments have proved that the selection of 35% ground mineral powder–fly ash as composite mineral admixture is most effective in ensuring the compressive strength and carrying strength of concrete.

The mixed admixture of fly ash and mineral powder can significantly enhance the crack resistance, corrosion resistance, and durability of high-performance concrete, as well as improve the impermeability.

The performance analysis of this thesis provides a useful discussion of high-performance concrete materials in civil construction; however, the depth and practicality of the research can be further enhanced by incorporating more performance indicators, abundant experimental data, in-depth mechanistic analysis, and case studies of practical applications. In addition, taking sustainability and future trends into account will also provide a more comprehensive perspective on the development of the high-performance concrete field.

## Figures and Tables

**Table 1:** Mix proportions of mineral powder–fly ash composite mineral admixture with different dosages.

Mixing Amount (%)	Amount of per Square Meter (kg/m^3^)						
	Cement (kg)	Fly Ash (kg)	Mineral Powder (kg)	Sand (kg)	Gravel (kg)	Water (kg)	Additive (kg)
0	470	0	0	500	1000	165	4.8
15	410	25	35	500	1000	165	4.8
30	350	50	70	500	1000	165	4.8
45	260	90	120	500	1000	165	4.8
60	180	120	170	500	1000	165	4.8

**Table 2:** Effect of compound mineral admixture amount on workability of concrete.

Mineral Powder-Fly Ash (%)	Slump (cm)	Expansion (cm)	30 min Slump Loss (cm)	60 min Slump Loss (cm)
100–0	16	34	5.4	7.4
60–40	19	40	3.2	5.2
50–50	23	53	2.5	4.6
40–60	23	55	2.5	4.6
0–100	21	55	2.1	4.1

**Table 3:** Influence of composite mineral admixtures on mechanical properties of concrete.

Mineral Powder-Fly Ash (%)	3 d Compressive Strength (MPa)	7 d Compressive Strength (MPa)	28 d Compressive Strength (MPa)	7 d Flexural Strength (MPa)	7 d Flexural Strength (MPa)	28 d Flexural Strength (MPa)
100–0	29	41	46	4.5	5.6	6.1
60–40	27	42	46	4.0	5.1	5.8
50–50	26	38	43	3.7	4.7	5.2
40–60	26	35	41	3.4	4.2	4.8
0–100	23	33	39	3.0	3.6	4.6

**Table 4:** Cracking of C50 high-performance concrete.

NO.	Cracking Time (h)	Average Cracking Area (mm^2^)	Number of Cracks per Unit Area (piece/m^2^)	Total Cracking Area per Unit Area (mm^2^/m^2^)
(Mixed with FMH + KF)	19	21	8	203
(Mixed with FMH)	14	30	11	300
(Mixed with KF)	10	124	16	1754
(Reference sample)	7	141	19	2250

**Table 5:** Sulfate corrosion resistance of C50 high-performance concrete.

NO.	28 d Corrosion Resistance Coefficient	7 d Corrosion Resistance Coefficient
(Mixed with FMH + KF)	88	89
(Mixed with FMH)	74	81
(Mixed with KF)	86	82
(Reference sample)	73	75

**Table 6:** Relative dynamic modulus of concrete under different freeze–thaw cycles.

NO.	Freeze Cycles (times)				
	50	100	150	200	250
	Relative Elastic Modulus (%)				
(Mixed with FMH + KF)	99	98	97	96	95
(Mixed with FMH)	95	94	90	88	85
(Mixed with KF)	91	88	83	79	76
(Reference sample)	84	81	78	70	51

**Table 7:** Impermeability of concrete.

Test Result	Reference Sample	(Mixed with KF)	(Mixed with FMH)	(Mixed with FMH + KF)
The water pressure of the third specimen when it seeps (MPa)	0.7	1.3	1.5	1.9
Water seepage grade	P5	P11	P13	P17

## Data Availability

The figures used to support the findings of this study are included in the article.
